# Supplementary material for: Social Box: Influence of a New Housing System on the Social Interactions of Stallions When Driven in Pairs
Source: Animals (Basel). 2022 Apr 21;12(9):1077. doi: 10.3390/ani12091077 (PMC9099530; doi:10.3390/ani12091077)
Supplement: Supplementary file 1 [file animals-12-01077-s001.zip › animals-1638846-supplementary.pdf]

Supplementary Table S1: Descriptive statistics for all observed stallion interactions per stallions. Total number of instances; mean ± SE; min-max; over the course of the study.

Behaviour	Total number of instances	Total number of instances; mean ± SE; min-max, range; over the course of the study for each horse									
		Horse 1 (n=12)	Horse 2 (n=10)	Horse 3 (n=9)	Horse 4 (n=12)	Horse 5 (n=12)	Horse 6 (n=11)	Horse 7 (n=8)	Horse 8 (n=12)	Horse 9 (n=43)	Horse 10 (n=43)
AH	2426	255	97	156	137	137	112	108	88	410	926
	14.1 ± 0.6	21.25 ± 1.6	9.7 ± 1.4	17.33 ± 1.8	11.42 ± 1.4	10.42 ± 1.81	10.18 ± 1.56	13.5 ± 1.92	7.33 ± 1.43	9.53 ± 0.69	21.53 ± 1.19
	0-39	10-31	4-18	11-26	6-21	0-23	3-18	8-23	1-19	0-19	4-39
BH	3	0	0	0	3	0	0	0	0	0	0
	0.02 ± 0.01	0	0	0	0.25 ± 0.18	0	0	0	0	0	0
	0-2	0	0	0	0-2	0	0	0	0	0	0
AS	4919	322	402	260	461	455	204	300	193	1200	1122
	28.60 ± 1.20	26.83 ± 2.45	40.2 ± 4.38	28.98 ± 4.69	38.42 ± 4.41	37.92 ± 4.54	18.55 ± 3.03	37.50 ± 6.90	16.08 ± 3.90	27.91 ± 2.59	26.09 ± 2.04
	2-89	11-42	12-61	13-56	24-77	16-65	5-35	23-83	2-51	7-89	10-66
RS	37	3	0	0	0	0	0	0	29	5	0
	0.22 ± 0.09	0.25 ± 0.25	0	0	0	0	0	0	2.42 ± 1.07	0.12 ± 0.08	0
	0-10	0-3	0	0	0	0	0	0	0-10	0-3	0
AM	2026	114	199	133	82	337	146	50	77	411	477
	11.78 ± 0.72	9.50 ± 1.62	19.9 ± 3.79	14.78 ± 2.37	6.83 ± 1.32	28.08 ± 4.59	13.27 ± 2.18	6.25 ± 1.22	6.42 ± 1.54	9.56 ± 1.07	11.09 ± 1.11
	0-69	3-21	5-35	4-26	0-16	14-69	4-25	1-11	1-20	0-27	2-35
RM	27	6	0	9	0	0	0	0	3	7	2
	0.16 ± 0.05	0.50 ± 0.50	0	1.00 ± 0.44	0	0	0	0	0.25 ± 0.25	0.16 ± 0.09	0.05 ± 0.03
	0-6	0-6	0	0-3	0	0	0	0	0-3	0-3	0-1
Total horse interactions	9438	700	698	558	683	929	462	458	390	2033	2527
	54.87 ± 1.68	58.33 ± 3.54	69.8 ± 7.45	62.00 ± 5.14	56.92 ± 4.56	77.42 ± 8.48	42.00 ± 4.62	57.25 ± 9.44	32.50 ± 4.70	47.28 ± 2.96	58.77 ± 3.00
	8-151	37-74	24-100	45-96	41-98	37-151	12-62	33-117	8-71	11-110	25-113
